# Hypomethylation of the CTCFL/BORIS promoter and aberrant expression during endometrial cancer progression suggests a role as an Epi-driver gene

**DOI:** 10.18632/oncotarget.1697

**Published:** 2014-01-28

**Authors:** Erling A. Hoivik, Kanthida Kusonmano, Mari K. Halle, Anna Berg, Elisabeth Wik, Henrica M. J. Werner, Kjell Petersen, Anne M. Oyan, Karl-Henning Kalland, Camilla Krakstad, Jone Trovik, Martin Widschwendter, Helga B. Salvesen

**Affiliations:** ^1^ Department of Obstetrics and Gynecology, Haukeland University Hospital, Bergen, Norway; ^2^ Centre for Cancer Biomarkers, Department of Clinical Science, University of Bergen, Bergen, Norway; ^3^ Computational Biology Unit, University of Bergen, Norway; ^4^ Centre for Cancer Biomarkers, Department of Clinical Medicine, University of Bergen, Bergen, Norway; ^5^ Department of Pathology, Haukeland University Hospital, Bergen, Norway; ^6^ Department of Microbiology, Haukeland University Hospital, Bergen, Norway; ^7^ Department of Women's Cancer, University College London Elizabeth Garrett Anderson Institute for Women's Health, University College London, United Kingdom

**Keywords:** CTCFL/BORIS, CTCF, metastasis, recurrence, epi-driver gene

## Abstract

Cancers arise through accumulating genetic and epigenetic alterations, considered relevant for phenotype and approaches to targeting new therapies. We investigated a unique collection of endometrial cancer precursor samples and clinically annotated primary and metastatic lesions for two evolutionary and functionally related transcription factors, CCCTC-binding factor (zinc finger protein) (CTCF) and its paralogue CTCF-like factor, also denoted Brother of the Regulator of Imprinted Sites (CTCFL/BORIS). CTCF, a chromatin modeling- and transcription factor, is normally expressed in a ubiquitous fashion, while CTCFL/BORIS is restricted to the testis. In cancer, CTCF is thought to be a tumor suppressor, while CTCFL/BORIS has been suggested as an oncogene. CTCF mutations were identified in 13 %, with CTCF hotspot frameshift mutations at p.T204, all observed solely in the endometrioid subtype, but with no association with outcome. Interestingly, CTCFL/BORIS was amongst the top ranked genes differentially expressed between endometrioid and non-endometrioid tumors, and increasing mRNA level of CTCFL/BORIS was highly significantly associated with poor survival. As aberrant CTCFL/BORIS expression might relate to loss of methylation, we explored methylation status in clinical samples from complex atypical hyperplasia, through primary tumors to metastatic lesions, demonstrating a pattern of DNA methylation loss during disease development and progression in line with the increase in CTCFL/BORIS mRNA expression observed. Thus, CTCF and CTCFL/BORIS are found to diverge in the different subtypes of endometrial cancer, with CTCFL/BORIS activation through demethylation from precursors to metastatic lesions. We thus propose, CTCFL/BORIS as an Epi-driver gene in endometrial cancer, suggesting a potential for future vaccine development.

## INTRODUCTION

Endometrial cancer (EC) of the *uterine corpus*, a common malignancy of the female genital tract, arises as a consequence of the accumulation of genetic and epigenetic alterations, under the influence of environmental and systemic factors [1-3]. For the majority of the patients, abnormal vaginal bleeding contributes to an early diagnosis, with the possibility to offer curative primary surgical treatment though restricted or extended surgical procedures [4]. Although overall survival rate is as high as 85 % at 5 years [1], patients with metastatic endometrial cancer have poor prognosis, a median survival of approximately one year, and no improvement on survival over the last decades [4].

Endometrial cancer is traditionally classified into type I and type II subtypes. Type I cancers account for 80-85 % of EC cases, are of endometrioid histology, more often well differentiated and associate with favorable prognosis. In contrast the type II cancers are non-endometrioid carcinomas, poorly differentiated and associate with poorer survival [1]. Although considerable overlap exists, the type I and II distinction is also supported by differences in molecular alterations: Microsatellite Instability (MSI), *PTEN, KRAS* and *CTNNB1* (encoding β-Catenin) mutations are often found in the type I, while *TP53* mutations and *ERBB2* (encoding HER-2) and MYC amplifications are more frequently detected in type II [1]. Also, loss of Estrogen (ERalpha) and Progesterone receptors (PR) is a feature of type II [4].

The CTCF CCCTC-binding factor (zinc finger protein) is a conserved transcription factor involved in gene regulation of a wide range of target genes, including the oncogene MYC and tumor suppressors such as *BRCA1* and *TP53* [5-8]. CTCF is also involved in chromosome “gymnastics”, by looping chromatin to ensure that regulatory *cis* regions are positioned correctly for transcriptional activity [9]. Moreover, CTCF function as an insulator protein in X-chromosome inactivation and imprinting, being important in developmental processes to define functional regions of the genome during mammalian development [10, 11]. CTCF has a paralogue in the CTCF-like factor, also denoted Brother of the Regulator of Imprinted Sites (CTCFL/BORIS) [12]. The two factors display distinct mutual exclusive expression patterns; CTCF is ubiquitously expressed, except in testis, while CTCFL/BORIS expression is specific to germ cells of the testis [12, 13]. The functional characteristics of CTCFL/BORIS are less explored than those of its counterpart CTCF, but a role in setting the re-methylation marks on the genome of male germ cells during spermatogenesis, as well as in testicular growth and male fertility has been supported based on studies of experimental knockout mice [12, 14, 15]. CTCFL/BORIS fall into the category of Cancer/Testis (CT) antigens (CTCFL/BORIS; CT27), a group of tumor associated genes aberrantly expressed in many cancers, but with restricted expression confined to the testis in their normal state, thus belonging to a group of genes suggested to represent targets for future cancer vaccine development [16, 17].

In the present study we have performed comprehensive molecular profiling of a unique sample collection of fresh frozen endometrial cancer precursor lesions in parallel with clinically annotated primary and metastatic endometrial carcinoma lesions to explore potential links between disease progression and *CTCF* mutations, aberrant expression of *CTCFL/BORIS* and *CTCFL/BORIS* DNA methylation. We report for the first time, that CTCF and CTCFL/BORIS seem to diverge in the different subtypes of endometrial cancer; with CTCF mutations occurring in endometrioid subtype and aberrant expression of CTCFL/BORIS being defined to cases of non-endometrioid subtype. Aberrant CTCFL/BORIS expression is induced through promoter hypomethylation in early steps of cancer progression, with increased mRNA expression from premalignant to primary tumors and further to metastatic lesions. This suggests CTCFL/BORIS as an Epi-driver gene with a potential for future vaccine development in endometrial cancer.

## RESULTS AND DISCUSSION

### CTCF p.T204 hotspot mutations found only in endometrioid tumors

**Figure 1:**
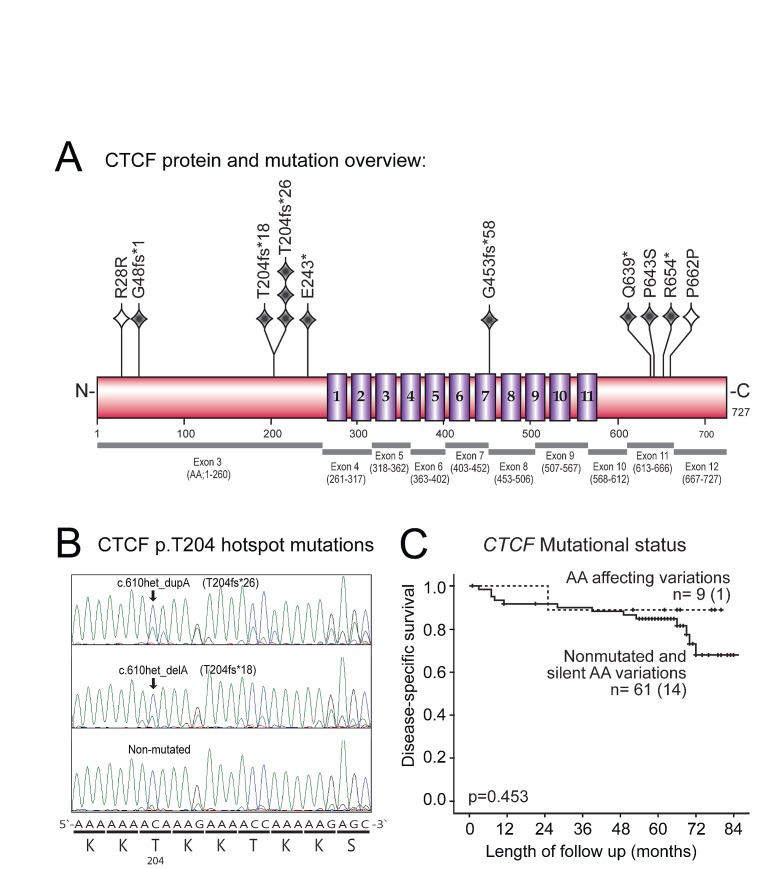
CTCF mutations, p.T204 hotspot site and association with disease-specific survival in primary endometrial carcinomas (A) Schematic overview of the CTCF protein showing position of mutations relative to Zink-finger domains (numbered 1-11). Grey diamonds indicate nonsense/frameshift mutation, open diamonds indicate silent mutation, one diamond per mutation. Exons corresponding to translated protein regions are shown below protein structure. CTCF gene; NCBI RefSeq NM_006565, chromosome position chr16:67596310-67673088 on GRCh37/hg19 assembly. (B) Chromatogram of the hotspot mutations (arrows) at position c.610, causing frameshift mutations at corresponding protein position p.T204. Consensus DNA nucleotide sequence along with protein sequence is shown below. (C) Disease-specific survival plot for patients with or without *CTCF* mutations indicates no significant differences in *CTCF* mutated vs. non-mutated group.

Based on the key importance of CTCF in chromatin organization and transcriptional regulation, together with accumulating reports identifying CTCF mutations in cancer patients, we performed sequencing of all coding exons of *CTCF* in 70 primary endometrial carcinomas, identifying a mutational frequency of 13 % (Figure 1A). A recent cross-cancer study, has pointed to *CTCF* mutations being most frequently found in endometrial cancer (16.5 %) across 12 major cancer types (overall frequency of 2.5 %) [18]. The Sanger Catalogue of Somatic Mutations in Cancer (COSMIC) database, presents a mutation rate of 17 % for endometrial cancer samples (n = 281) compared to 1.7 % mutation rate across 43 cancer types explored (132 mutated in 7928 explored samples (Sanger database August 2013) [19]. Thus, we find a comparable and high frequency of CTCF mutations in endometrial carcinomas in line with the TCGA (The Cancer Genome Atlas) study and the Sanger database.

A recent study on patients with developmental disorders and intellectual disability, suggests a functional link between CTCF mutations and cognitive processes [20]; in contrast a functional descriptive link of CTCF mutations towards cancer is lacking. In our study, a cluster of four mutations was found in the C-terminal of the protein at AA positions 639-662, as well as a hotspot mutation site at p.T204 causing frameshift in four cases predicting severe impact on function (p.T204fs*18, p.T204fs*26; Fig 1A, B and Supplementary Table S1). Interestingly, a confirmation of the recurrent mutational site at p.T204 in endometrial cancer was published while our study was ongoing, reporting that this particular slippage mutation was frequent in MSI positive tumors [21]. In addition, both the Sanger database and the TCGA based pan cancer study suggest p.R377 as another hotspot mutational site, although we did not see this in our analysis [22].

It is interesting that our study and the TCGA data both reveal CTCF mutated cases present only in the endometrioid subtype. Although not reaching statistically significance in our data, the larger pan cancer study [18] confirms this as a statistically significant correlation, with no mutated cases in the serous/mixed classes (Supplementary Table S1 and Figure S1), although a single exception has been reported [23]. Despite this, CTCF mutations seem to be limited to cases with the lesser aggressive endometrioid histology.

Neither CTCF mutations, nor CTCF mRNA expression data, did significantly influence survival or associate with other clinico-pathological markers for outcome (Figure 1B, Supplementary Table S2-3). This was consistent with mutational and clinical data downloaded from TCGA [24].

### CTCFL/BORIS is differentially expressed in endometrioid compared to non-endometrioid tumors

In light of the finding that all CTCF mutations were solely confined to the endometrioid subtype, we performed an analysis of significantly differently expressed genes (SAM, Significance analysis of microarrays [25]) between endometrioid vs. non-endometrioid subtypes. We discovered the CTCF-related factor named CTCFL/BORIS to be significantly increased in the non-endometrioid subtype (Among the top 417 genes [not shown] with estimated False Discovery Rate [FDR] < 0.001, q-value < 0.001, and fold change ≥ 2.000, CTCFL/BORIS was ranked 24.^th^ with fold change of 2.257). Since the CTCFL/BORIS, closely related to CTCF, has a potential role in methylation, and belongs to a group of tumor associated genes (CT antigens) aberrantly expressed in many cancers, but with normal expression restricted to the testis, we were prompted to further investigate this factor in relation to tumor progression and clinical phenotypes.

### High CTCFL/BORIS level identifies aggressive endometrial carcinoma

**Figure 2:**
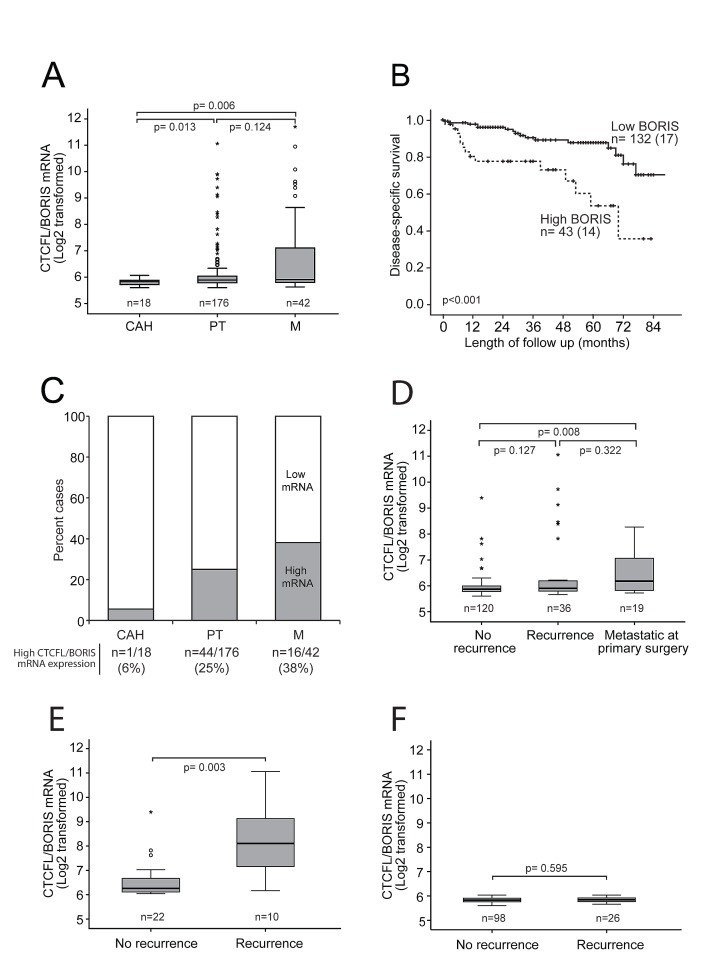
Increased CTCFL/BORIS mRNA expression level associates with cancer progression and poor survival (A) *CTCFL/BORIS* mRNA expression increases significantly through the stages of cancer development and progression from complex atypical hyperplasia (CAH) to primary tumors (PT) to metastases (M). (B) High *CTCFL/BORIS* mRNA expression level (above upper quartile) identifies endometrial cancer patients with poor disease-specific survival. (C) Bar graph presentation showing proportions of cases with high *CTCFL/BORIS* expression (above upper quartile) for the stages of cancer development and progression from CAH to PT to M. (D) Box plot presentation of *CTCFL/BORIS* mRNA levels in relation to systemic disease, i.e. development of recurrence or presence of non-resectable metastatic disease at primary surgery. (E, F) Box plot presentation of *CTCFL/BORIS* mRNA expression in relation to development of recurrent disease stratified for expression levels in primary tumors; compare high expression (E) to low (F) expression. A significant association between *CTCFL/BORIS* mRNA level and recurrent disease is seen only for the patient group with initial high *CTCFL/BORIS* mRNA level in primary lesions (E).

*CTCFL/BORIS* mRNA expression increases significantly through the stages of cancer development investigated, from complex atypical hyperplasia (CAH) to primary tumors (PT) and metastatic lesions (M) (Figure 2A and C). All the clinically established markers for aggressive endometrial carcinoma including high age, high FIGO stage, non-endometrioid histology, high grade and hormone receptor loss were significantly associated with high *CTCFL/BORIS* mRNA levels as listed in Table 1. In line with this, the group of patients with high *CTCFL/BORIS* mRNA expression level, defined by the upper quartile, has a 5 year disease specific survival of 50 % compared to 90 % for the reminder (Figure 2B). To our knowledge this describes for the first time the aberrant expression of CTCFL/BORIS across these cancer developmental stages, and suggests the potential of CTCFL/BORIS as both a biomarker as well as a highly relevant target in metastatic endometrial cancer disease in particular.

**Table 1:** Clinico-pathological data in relation to CTCFL/BORIS mRNA expression in endometrial carcinomas

Variable^b^	Category	CTCFL/BORIS Low n (%)	CTCFL/BORIS High n (%)	p-value^a^
Age	≤66	71 (85)	13 (15)	0.009
	>66	61 (66)	31(34)	
FIGO stage	I	100 (80)	26 (21)	0.001
	II	12 (92)	1 (8)	
	III	16 (67)	8 (33)	
	IV	4 (31)	9 (69)	
Histological subtype	Endometrioid	119 (84)	23 (16)	<0.001
	Non-endometrioid	13 (38)	21 (62)	
Histological grade	Grade 1-2	86 (85)	15 (15)	<0.001
	Grade 3	45 (62)	28 (38)	
ERα (IHC)	High	102 (81)	24 (19)	0.020
	Low	26 (62)	16 (38)	
PR (IHC)	High	104 (83)	22 (18)	0.001
	Low	25 (56)	20 (44)	

a Pearson Chi-Square exact significance test, two-sided.

b Data missing (n patients); Histological grade (2), ERα (8), PR (5).

Interestingly, *CTCFL/BORIS*, normally not expressed in the endometrium, was found to increase during cancer development and progression, which was also reflected in poor survival for patients with high expression levels. Samples from patients with no recurrence tended to have lower CTCFL/BORIS level compared to those who did recur (p-value of 0.127; Figure 2D), while primary tumors with metastatic disease at the time of primary surgery displayed a significantly higher level of *CTCFL/BORIS* (p-value of 0.008). Stratifying patients according to low vs. high *CTCFL/BORIS* levels, a significant higher expression level of *CTCFL/BORIS* was seen for recurrent cases only for the high expression group (p-value 0.003; Figure 2E and 2F).

### DNA methylation index (MI) of CTCFL/BORIS promoter decreases from premalignant to malignant lesions

**Figure 3:**
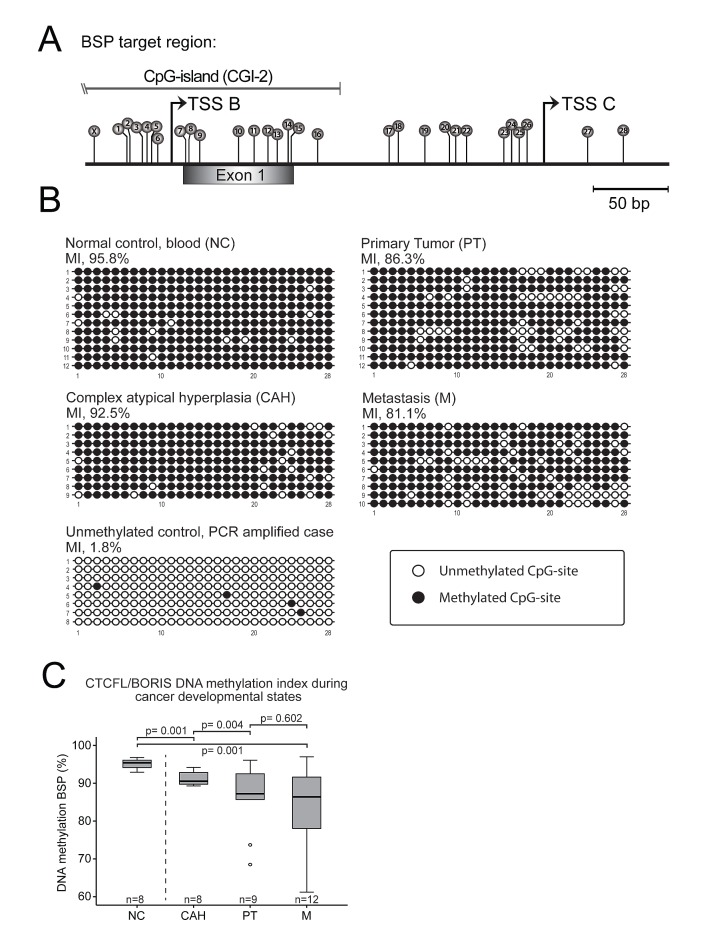
DNA methylation index (MI) shows decreasing level of CTCFL/BORIS promoter methylation from premalignant through primary to metastatic endometrial carcinoma lesions (A) Overview of CpG-sites marked as lollipops numbered 1-28, on *CTCFL/BORIS* promoter targeted in the bisulphite sequencing assay. Site marked X not included due to integration in PCR primer. The target region contains two dominant transcription start sites: TSS B and TSS C and exon 1 of the gene (extended overview in Figure S2). (B) Representative bisulphite sequencing samples of normal control (NC), complex atypical hyperplasias (CAH), primary tumors (PT) and metastasis (M). The *CTCFL/BORIS* promoter shows a gradual loss of methylation through cancer developmental stages compared to normal control (from blood buffy coat). Each horizontal line represents an epiallele, with each individual CpG-site shown as either methylated CpG sites (black circles) or unmethylated CpG site (open circles), with positions corresponding to numbering in A. The vertical stack of epialleles describes analysis of multiple colonies/clones. The controls showed a high degree of methylation of mean value of 95.1 %, while an *in vitro* PCR generated unmethylated control was 1.8 % methylated, indicating robust analysis. Analysis close to mean values in C) was selected for display. (C) Comprehensive box-plot of MI-values significantly declining through cancer progression with mean values for normal controls of 95.1 %, hyperplasias with atypia 91.2 %, primary tumors 86.2 % and metastases 83.8 %.

We then hypothesized that the aberrant *CTCFL/BORIS* expression might relate to loss of methylation as irregular CTCFL/BORIS expression is found in a range of tumorigenic cell lines and in several cancers including liver, breast, ovarian, prostate, colon, lung, endometrial cancers as well as melanoma and glioblastoma [26-28]. One possible mechanism of the increased CTCFL/BORIS expression might be due to loss of epigenetic traits during cancer development/progression, a well-established element in inducing aberrant CT antigen expression in somatic tissues [17, 29, 30]. We therefore characterized DNA methylation status of the CTCFL/BORIS promoter, spanning across transcriptional start sites of alternative B- and C- promoters [14, 28] by bisulphite sequencing analysis on tissues representing various stages of cancer development (Figures 3A and S2A). Complex atypical hyperplasias, primary tumors and metastatic samples were compared to normal control (NC, buffy coat fraction from blood samples). As the fine mapping of the methylation status of the single CpG-sites within the target did not reveal a distinct demethylation pattern, (Figure 3B), a methylation index (MI) was calculated for each specimen and demonstrated a significant reduction in the DNA methylation level during the stages of cancer development, most pronounced for the step from complex atypical hyperplasias to primary tumors. Although not reaching statistical significance, the metastatic sample set showed a tendency to less CTCFL/BORIS promoter methylation compared to primary tumors exploring samples investigated by k27 array (Illumina Infinium Human Methylation27 BeadChip array) (Supplementary Figure S2C) also inversely mirroring mRNA expression levels (Figure 2A). There was a significant correlation between the results from the MI and available data for k27 assay for overlapping samples (Supplementary Figure S2C). Taken together, the methylation pattern observed appears to be in line with the general notion that hypomethylation occurs as an early step in cancer [30].

### High CTCFL/BORIS level and loss of CTCFL/BORIS promoter methylation associate with aggressive subgroups in external data

We further explored expression levels and methylation pattern for *CTCFL/BORIS* in TCGA data for primary tumors for the methylation assay k27 and Agilent 244k custom gene expression microarray. We validated a similar significant correlation between high *CTCFL/BORIS* mRNA expression and non-endometrioid and grade 3 subgroups in the TCGA data (Supplementary Figure S3A). Interestingly, in the TCGA data we also find a significant correlation between *CTCFL/BORIS* hypomethylation and non-endometrioid histology and grade 3 inversely mirroring mRNA expression levels (Supplementary Figure S3B).

### Hormone receptor loss associates with high CTCFL/BORIS expression

Selected genes found to be highly differentially expressed comparing CTCFL/BORIS low vs. high mRNA microarray expression levels in primary tumors, is presented in Table 2 and as a full list in Supplementary Table S4. Interestingly, genes encoding estrogen- (ESR1), progesterone- (PGR) and androgen (AR) receptors were all down-regulated in the group expressing *CTCFL/BORIS* at high mRNA levels (defined by upper quartile limit). This fits well with the long time reported association between loss of ERalpha/PR expression and aggressive disease [31, 32]. Androgen receptor has been found to have anti-proliferative effects in the normal endometrium, and therefore loss of functional AR might promote carcinogenesis [33].

**Table 2:** Selected genes differentially expressed in groups based on low and high CTCFL/BORIS expression

Regulation	Gene Name	Description	Fold Change
UP^a^	IGF2BP1	**Insulin-like growth factor 2 mRNA-binding protein 1**	2.529
	IGF2BP2	**Insulin-like growth factor 2 mRNA-binding protein 2**	2.620
	SOX11	SRY (sex determining region Y)-box 11	2.343
DOWN^b^	ESR1	Estrogen receptor α	3.501
	PGR	Progesterone receptor	2.993
	AR	Androgen receptor	2.067
	SOX17	SRY (sex determining region Y)-box 17	2.180

a Increased expression of genes in the group defined to having high CTCFL/BORIS mRNA expression (according to upper quartile limits), compared to the group of low CTCFL/BORIS mRNA expression.

b Reduced expression of genes in the group defined to having high CTCFL/BORIS mRNA expression, compared to the group of cases classified with low CTCFL/BORIS mRNA expression.

### CTCFL/BORIS - a potential Epi-driver gene in endometrial cancer?

A study investigating the genome-wide CTCF and CTCFL/BORIS binding to chromatin in ChIP(Chromatin ImmunoPrecipitation)-experiments in cell lines, reveals that CTCFL/BORIS almost exclusively binds to CTCF consensus sites at promoters suggesting an important regulatory function [15]. Extrapolating this to cancer proposes that aberrant expression of CTCFL/BORIS might overrule genes normally regulated by CTCF, or at least compete with CTCF on transcriptional target genes with different outcome in regulation due to differences in N- and C-terminals and discrete protein interaction partners [12-15, 34, 35]. Exogenous expression of CTCFL/BORIS in cell systems induced the expression of hTERT, a subunit of the telomerase enzyme conferring immortality in tumorigenesis, suggesting one mechanism through which CTCFL/BORIS may lead to increase in proliferation in cancer [36].

The concept of Epi-driver genes is fairly new, and we propose for the first time *CTCFL/BORIS* as an potential Epi-driver gene according to the criteria suggested by Vogelstein [37] based on the following; Epigenetic alterations characterized by promoter hypomethylation lead to aberrant expression of *CTCFL/BORIS* mRNA expression level in cancer, cancerous expression is clearly associated with clinical phenotypes and cancer progression, it is not frequently mutated and its related factor CTCF (analogous to CTCFL/BORIS) has recently been suggested to be a Mut-driver gene in endometrial cancer [18, 38].

There are several aspects of Epi-driver genes that are potentially beneficial in therapeutic approaches. First, as epigenetic mechanisms are inducing aberrant expression of Epi-driver genes, it also suggests that future specific epigenetic modulator drugs might be able to target specific epigenetic changes causing this, as epigenetic changes are potentially reversible [39, 40]. Second, it also suggests that Epi-drivers might be targeted by cancer vaccines, as has been suggested for Cancer/Testis antigens [17]. In fact, through a metastatic mammary mouse model, a BORIS-based cancer vaccine delivered by dendritic cells was shown to be effective in inhibiting tumor growth and the formation of metastasis [41]. Clinical trials targeting CT genes are few, but NY-ESO-1 (New York esophageal squamous cell carcinoma 1, also known as CTAGB1, CT6.1) was targeted by adoptive immunotherapy employing genetically modified lymphocytes in patients with metastatic synovial cell sarcoma or melanoma patients and was shown to be effective with impressive responses to treatment with no apparent toxicity effect (Effective responses of 67 % and 45 %) [42]. Indeed, the idea of targeting the testis-specific genes normally not expressed in females, but in systemic endometrial carcinomas, through cancer vaccines, is appealing.

## MATERIAL AND METHODS

### Patient material ethical approvals

Patients were included in the study after written informed consent, approved by the Norwegian Data Inspectorate, Norwegian Social sciences Data Services, the Regional Research Ethics Committee in Medicine and the local Hospital (NSD15501; REK 052.01). Samples were collected from patients treated at the Haukeland University Hospital, Norway, as previously described [31]. Tumors were snap frozen in liquid nitrogen (l) and stored at minus 80°C until validation of > 50 % purity for the malignant epithelial component before RNA or DNA extraction. Blood (buffy coat) was collected from normal healthy individuals, age matched to the EC group of patients, for DNA extraction to be used as control samples for the DNA methylation analyses.

### RNA extraction and RNA analysis

RNA was extracted with the RNAeasy tissue kit from Qiagen, following the standard protocol as recommended. Cy3-cRNA labeled samples were hybridized to Agilent Whole Human Genome Microarrays 44k array (Cat.nr.G4112F) according to the manufacturers' instructions, and scanned employing the Agilent Microarray Scanner Bundle as previously described [31]. The arrays were quantile normalized using median spot signal, log2 transformed. Statistical analysis of differentially expressed genes (SAM) was performed with the J-Express software [25]. The cutoff levels for low- and high *CTCFL/BORIS* mRNA expression was determined based on quartile limits merging groups with similar survival in Kaplan-Meier survival analysis. In this, as well as support from the CTCFL/BORIS literature [14, 43], we assume that cases classified within the group of low *CTCFL/BORIS* mRNA expression, do not express CTCFL/BORIS or alternatively at low levels, although we did not perform additional RT-PCR analysis to confirm this.

### DNA extraction, sequencing and methylation analysis

Genomic DNA (gDNA) was extracted after proteinase K digestion, and precipitated following a standard protocol. gDNA was bisulphite treated, amplified and hybridized employing the Illumina Infinium HumanMethylation27 BeadChip kit (see [44] for details). For sequencing, 10 ng gDNA was whole genome amplified by employing the GenomePlex Complete Whole Genome Amplification (WGA) kit (Sigma) with the GenElute PCR Clean-Up kit (Sigma) for purification. Amplicons for direct sequencing were obtained by PCR with 25 ng WGA-material with HotSTAR polymerase (Qiagen) and primers according to Supplementary Table S5. After agarose-gel purification on Qiagen gel-extraction kit, amplicons (all coding exons of CTCF) were sequenced by universal M13-primers utilizing the BigDye Terminator Sequencing Kit, version 1.3, analyzed on an ABI Prism 3100 genetic analyzer (Applied Biosystems). Sequences were inspected with Sequence Scanner software 1.0 (Applied Biosystems), excluding variants present in the SNP database (dbSNP) http://www.ncbi.nlm.nih.gov/snp). Mutational data were compared against the Sanger COSMIC database (http://www.cancer.sanger.ac.uk/cancergenome/projects/cosmic/), as well as TCGA data (http://www.cancergenome.nih.gov). Methylation analysis of CTCFL/BORIS NCBI RefSeq NR_072975, spanning nucleotides chr20:56099842-56100217 on GRCh37/hg19 assembly was performed in bisulphite sequencing assay. 200 ng of gDNA was treated in DNA methylation Direct kit by Zymo Research, following PCR by specific primers (available upon request) employing HotSTAR polymerase (Qiagen), and subcloning in pGEM-T-easy vector (Promega). Up to 12 colonies were sequenced from each tumor/case for the analysis of CpG-site specific DNA methylation in the CTCFL/BORIS promoter regions. Methylation positive and methylation depleted control PCR products (primers available upon request) spanning the CTCFL/BORIS promoter region were gel purified and in vitro methylated in the presence or absence of the SssI CpG methylase (New England Biolabs, United Kingdom) at 37°C for four hours with SssI (2 U/μg DNA) in presence of S-adenosylmethionine (SAM) (160 μM), with additional SssI (0.3 U/μg DNA) and SAM (160 μM) after two hours. Methylation indexes (MI) were calculated as a percentage of the methylated CpGs compared to the total CpGs in each analysis. For matters related to the Illumina Infinium Human Methylation27 BeadChip array, it was performed as described in reference [44].

### Statistical analysis

The Fischer exact tests was used to test the significance of associations between mutations within subtypes, employing the SPSS 18.0 statistical software. Alternatively, when appropriate, Pearson Chi-square exact two-sided tests were employed. All statistical tests were considered significant when p-values ≤ 0.05. Univariate survival analyses of time to death due to endometrial carcinoma (disease specific survival) were performed using the Kaplan-Meier method. Entry date was the date of primary surgery. Patients who died from other causes were censored at the date of death. Differences in survival between groups were estimated by two-sided log-rank (Mantel Cox) tests.

## SUPPLEMENTARY FIGURES AND TABLES




