# Supplementary material for: Hypomethylation of the CTCFL/BORIS promoter and aberrant expression during endometrial cancer progression suggests a role as an Epi-driver gene
Source: Oncotarget. 2014 Jan 28;5(4):1052–61. doi: 10.18632/oncotarget.1697 (PMC4011582; doi:10.18632/oncotarget.1697)
Supplement: Supplementary file 1 [file oncotarget-05-1052-s001.pdf]

Hypomethylation of the CTCFL/BORIS promoter and aberrant expression during endometrial cancer progression suggests a role as an Epi-driver gene – Hoivik et al

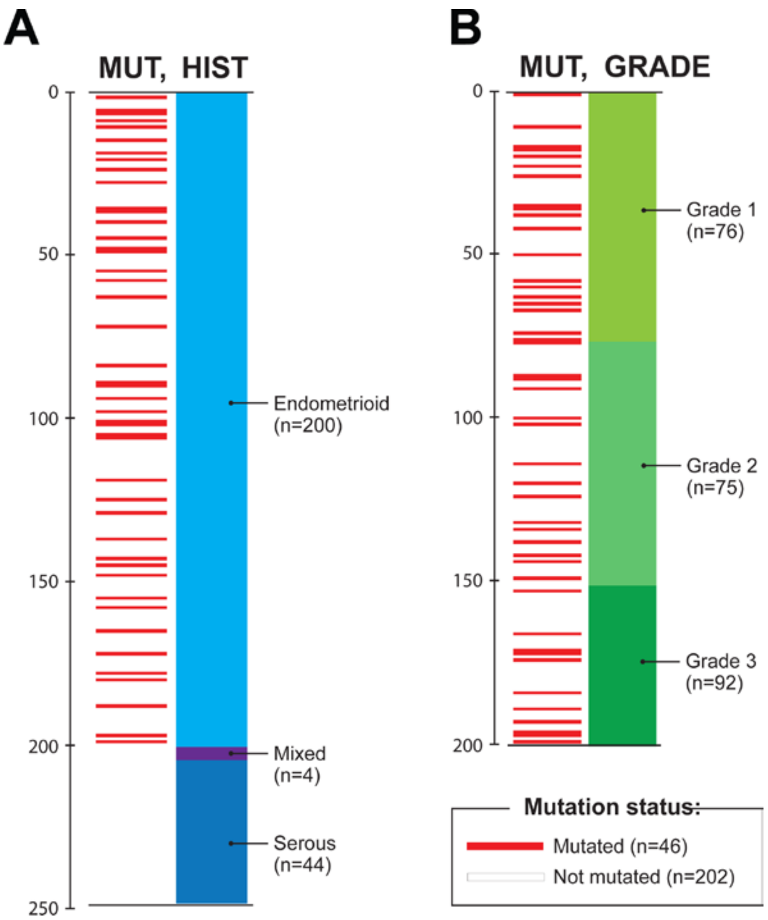

Figure S1: TCGA data of *CTCF* mutations in endometrial cancer.

Data specific for endometrial cancer and CTCF mutational status were downloaded from UCSC Cancer Genomics Browser (28) (Sept 2013). *CTCF* mutations in the TCGA data were reported solely in the endometrioid subtype as illustrated in (A). Distribution of *CTCF* mutations for endometrioid cases are shown in (B) demonstrating that the CTCF mutations are found across all histological grades in dataset from TCGA. Dataset details: TCGA uterine corpus endometrioid carcinoma (UCEC) somatic mutation, version 2013-05-19. Number of samples = 248.

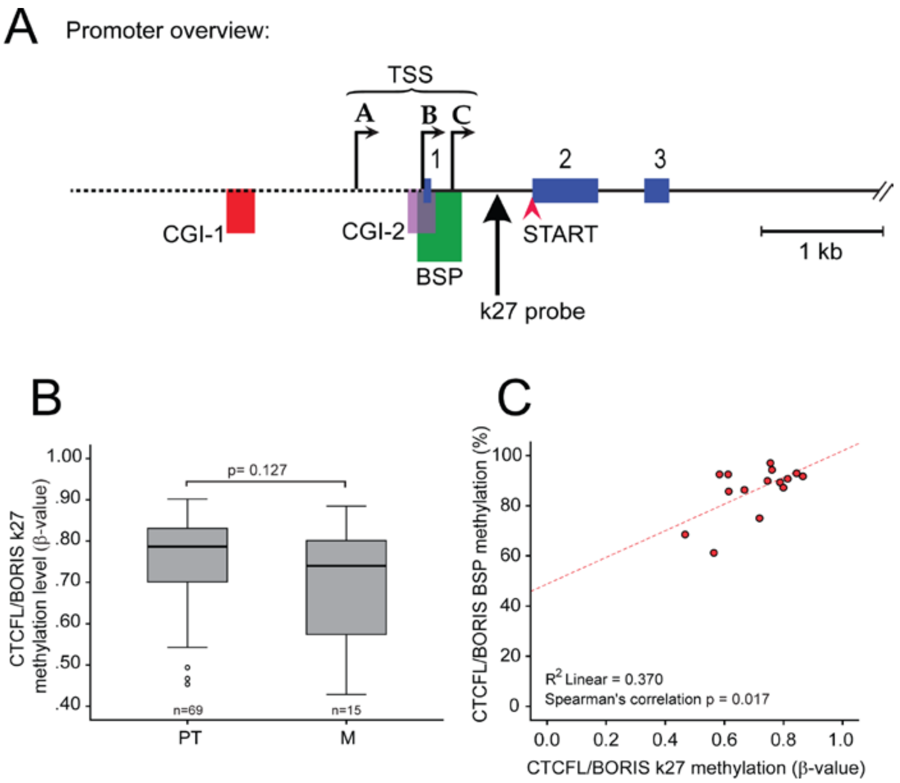

Figure S2: Overview of *CTCFL/BORIS* promoter region and k27 DNA methylation values

(A) Schematic overview of the 5`region of *CTCFL/BORIS*, including promoter, exons 1-3, CpG-islands (CGI-1, 2) and alternative transcription start sites (TSS A, B and C). Note the selected region for BSP analysis, targeting the potentially more appropriate region compared to the k27 probe (methylation analysis by Illumina Infinium HumanMethylation27 [k27] BeadChip platform, probe cg14264994 at chr20: 55532954) for *CTCFL/BORIS*. (B) Box-plot showing the β-values (21) of the *CTCFL/BORIS* k27 methylation probe showing a tendency of hypomethylation comparing primary tumors (PT) and metastatic lesions (M), although borderline significant (p-value 0.127). (C) Results assessing methylation status by the k27 methylation array and BSP analysis show good correlation for samples subjected to

investigation by both assays, supporting that BSP-based MI-values provides similar results as the k27 assay.

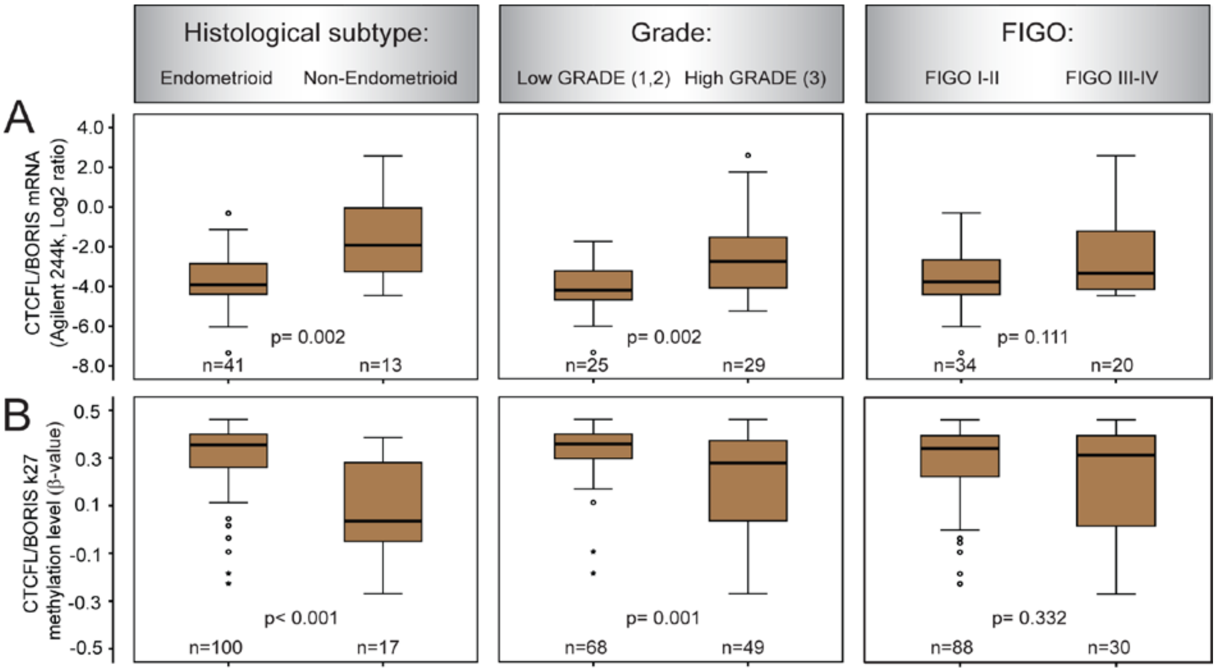

Figure S3: Array-based data of *CTCFL/BORIS* mRNA expression and DNA methylation levels in primary endometrial carcinomas from the TCGA data validate a significant correlation between high mRNA expression levels and non-endometrioid and grade 3 histologies (A). Also, a similar pattern for significant correlation between *CTCFL/BORIS* hypomethylation and none-endometrioid histology and grade 3 differentiation is observed (B). TCGA data (level 3-data, processed at UCSC) for CTCF/BORIS were downloaded as previously described (28) for Agilent 244k assay of mRNA expression (A) and Illumina k27 human methylation assay (B). Dataset details; TCGA uterine corpus endometrioid carcinoma (UCEC); (A) Gene expression label: AgilentG4502A_07_3 array, ID: TCGA_UCE C_ G 4 5 02A_07_3. Number of samples = 54. (B) Methylation label: HumanMethylation27, ID: TCGA_UCEC_hMethyl27. Both datasets downloaded by version 2013-09-07. Number of samples = 118.
