# Supplementary material for: Hypomethylation of the CTCFL/BORIS promoter and aberrant expression during endometrial cancer progression suggests a role as an Epi-driver gene
Source: Oncotarget. 2014 Jan 28;5(4):1052–61. doi: 10.18632/oncotarget.1697 (PMC4011582; doi:10.18632/oncotarget.1697)
Supplement: Supplementary file 2 [file oncotarget-05-1052-s002.docx]

**Supplementary Table S1: CTCF mutations in primary tumors of endometrial cancers of endometrioid subtype**

| **EC case** | **Mutated exon** | **CTCF nucleotide change^a^** | **Predicted CTCF amino acid change^b^** | **Predicted effect on CTCF protein** | **Mutation type** |
| --- | --- | --- | --- | --- | --- |
| a | Exon 3 | c.[=,84C>T] | p.28R>R | Silent | Silent |
| b | Exon 3 | c.[=,143dupG] | p.48G>fs*2 | Frameshift insertion | Duplication |
| c | Exon 3 | c.[=,610delA] | p.204T>fs*18 | Frameshift deletion | Deletion |
| d | Exon 3 | c.[=,610dupA] | p.204T>fs*26 | Frameshift insertion | Duplication |
| e | Exon 3 | c.[=,610dupA] | p.204T>fs*26 | Frameshift insertion | Duplication |
| f | Exon 3 | c.[=,610dupA] | p.204T>fs*26 | Frameshift insertion | Duplication |
| g | Exon 3 | c.[=,727G>T] | p.E243* | Nonsense | Premature STOP codon |
| h | Exon 8 | c.[=,1358delG] | p.453G>fs*58 | Frameshift insertion | Duplication |
| i | Exon 11 | c.[=,1915C>T] | p.639Q>* | Nonsense | Premature STOP codon |
| i | Exon 11 | c.[=,1927C>T] | p.643P>S | Missense | AA substitution |
| j | Exon 11 | c.[=,1960C>T] | p.654R>* | Nonsense | Premature STOP codon |
| g | Exon 11 | c.[=,1986C>G] | p.662P>P | Silent | Silent |
| k | Exon 12 | c.[=,*29T>G] | none | Noncoding | 3`UTR |

a Based on NM_006565. = indicates normal allele. ^b^Changes: fs; frameshift, *; stop codon.

n = 70 total, 10 AA affecting mutations in 9 cases, percent mutated total: 9/70 = 12.9 %.

None of these mutations has previously been deposited in the Sanger database.

**Supplementary Table S2: Clinico-pathological data in relation to CTCF mutations in endometrial carcinomas**

| **Variable^b^** | **Category** | **CTCF mutated  n (%)** | **Non-mutated  n (%)** | **p-value^a^** |
| --- | --- | --- | --- | --- |
| **Age** | ≤66 | 29 (83) | 6 (17) | 0.477 |
|  | >66 | 32 (91) | 3 (9) |  |
| **FIGO stage** | I | 49 (85) | 9 (15) | 0.697 |
|  | II | 2 (100) | 0 (0) |  |
|  | III | 3 (100) | 0 (0) |  |
|  | IV | 7 (100) | 0 (0) |  |
| **Histological subtype** | Endometrioid | 52 (85) | 9 (15) | 0.351 |
|  | Non-endometrioid | 9 (100) | 0 (0) |  |
| **Histological grade** | Grade 1-2 | 43 (88) | 6 (12) | 1.000 |
|  | Grade 3 | 18 (90) | 2 (10) |  |
| **ERα (IHC)** | High | 46 (85) | 8 (15) | 0.677 |
|  | Low | 12 (92) | 1 (8) |  |
| **PR (IHC)** | High | 42 (84) | 8 (16) | 0.427 |
|  | Low | 18 (95) | 1 (5) |  |

^a^Pearson Chi-Square exact significance test, two-sided.

^b^Data missing (n patients); Histological grade (1), ERα (3), PR (1).

**Supplementary Table S3: Clinico-pathological data in relation to CTCF mRNA expression**

| **Variable^b^** | **Category** | **CTCF mRNA  median** | **CTCF mRNA**  **Std Deviation** | **n cases** | **p-value^a^** |
| --- | --- | --- | --- | --- | --- |
| **Age** | ≤66 | 9.27 | 0.70 | 84 | 0.066 |
|  | >66 | 9.13 | 0.77 | 92 |  |
| **FIGO stage** | I | 9.24 | 0.06 | 126 | 0.152 |
|  | II | 9.01 | 0.11 | 13 |  |
|  | III | 9.03 | 0.11 | 24 |  |
|  | IV | 9.27 | 0.23 | 13 |  |
| **Histological subtype** | Endometrioid | 9.23 | 0.05 | 142 | 0.156 |
|  | Non-endometrioid | 9.04 | 0.13 | 34 |  |
| **Histological grade** | Grade 1-2 | 9.24 | 0.07 | 101 | 0.442 |
|  | Grade 3 | 9.14 | 0.08 | 73 |  |
| **ERα (IHC)** | High | 9.19 | 0.06 | 126 | 0.532 |
|  | Low | 9.49 | 0.81 | 42 |  |
| **PR (IHC)** | High | 9.19 | 0.06 | 126 | 0.328 |
|  | Low | 9.28 | 0.11 | 45 |  |

^a^Pearson Chi-Square exact significance test, two-sided.

^b^Data missing (n patients); Histological grade (2), ERα (8), PR (5).

**Supplementary Table S4: Genes differentially expressed comparing CTCFL/BORIS mRNA expression (low v.s. high) groups^a^**

| **Upregulated in CTCFL/BORIS_High** | | | **Downregulated in CTCFL/BORIS_High** | | |
| --- | --- | --- | --- | --- | --- |
| **Gene number** | **Gene Name** | **Fold Change** | **Gene  number** | **Gene Name** | **Fold Change** |
| 1 | CTCFL/BORIS | 2.547 | 1 | PPAP2C | 2.895 |
| 2 | TP53TG3 | 2.106 | 2 | SPDEF | 4.398 |
| 3 | RYR1 | 2.136 | 3 | ESR1 | 3.501 |
| 4 | BM928667 | 2.362 | 4 | AL137566 | 5.086 |
| 5 | IGF2BP1 | 2.529 | 5 | PGR | 2.993 |
| 6 | A_32_P232647 | 2.398 | 6 | KIAA1324 | 5.700 |
| 7 | SALL4 | 2.661 | 7 | SLC40A1 | 2.564 |
| 8 | LRRN1 | 2.404 | 8 | SPECC1 | 2.046 |
| 9 | TUBB2B | 3.688 | 9 | SERINC2 | 2.023 |
| 10 | AK025975 | 2.077 | 10 | TFF3 | 5.653 |
| 11 | VCX | 2.700 | 11 | PRR15 | 2.578 |
| 12 | ENST00000330490 | 2.001 | 12 | CEACAM1 | 2.448 |
| 13 | LOC653216 | 2.062 | 13 | CAPS | 3.604 |
| 14 | VCX2 | 2.762 | 14 | MLPH | 3.513 |
| 15 | UCHL1 | 4.383 | 15 | TMEM16A | 2.530 |
| 16 | VCX3A | 2.737 | 16 | AGR2 | 3.977 |
| 17 | SLC6A13 | 2.059 | 17 | HGD | 3.510 |
| 18 | FAM77C | 2.188 | 18 | GCNT3 | 3.434 |
| 19 | A_23_P121234 | 2.421 | 19 | FAM110C | 2.622 |
| 20 | TCF15 | 2.233 | 20 | GALNT4 | 2.162 |
| 21 | C10orf114 | 2.072 | 21 | DEPDC6 | 2.117 |
| 22 | PDE6B | 2.076 | 22 | LRG1 | 2.378 |
| 23 | LIX1 | 2.368 | 23 | UBXD3 | 2.205 |
| 24 | MDFI | 2.085 | 24 | EYA2 | 3.181 |
| 25 | CLDN6 | 2.220 | 25 | TMC5 | 2.833 |
| 26 | SOX11 | 2.343 | 26 | AK026517 | 2.657 |
| 27 | ROBO3 | 2.199 | 27 | NT5E | 2.599 |
| 28 | PTX3 | 2.193 | 28 | GGTA1 | 2.214 |
| 29 | CR615016 | 2.136 | 29 | PIGR | 4.885 |
| 30 | PLAG1 | 2.198 | 30 | SOX17 | 2.180 |
| 31 | BX111592 | 2.238 | 31 | FLJ10847 | 3.251 |
| 32 | IGF2BP2 | 2.620 | 32 | GLYATL2 | 2.761 |
| 33 | RNF182 | 2.267 | 33 | UPK1B | 3.320 |
| 34 | SYT13 | 2.552 | 34 | KIAA1189 | 2.128 |
| 35 | BEX2 | 2.474 | 35 | ENTPD3 | 2.178 |
| 36 | HOXA4 | 2.166 | 36 | ATP2C2 | 2.570 |
| 37 | BMP7 | 2.969 | 37 | CA12 | 2.352 |
| 38 | SNIP | 2.030 | 38 | C9orf61 | 2.441 |
| 39 | NPTX2 | 2.014 | 39 | THC2736540 | 3.216 |
| 40 | DLK1 | 2.001 | 40 | THC2591546 | 2.957 |
| 41 | TNNT1 | 3.830 | 41 | ST6GALNAC1 | 2.846 |
| 42 | COL23A1 | 2.091 | 42 | IL20RA | 2.127 |
| 43 | NXF2 | 2.011 | 43 | SLPI | 2.153 |
| 44 | CRABP1 | 2.203 | 44 | ANKRD35 | 2.257 |
|  |  |  | 45 | ASRGL1 | 2.362 |
|  |  |  | 46 | HLA-DMB | 2.072 |
|  |  |  | 47 | TSPAN1 | 2.376 |
|  |  |  | 48 | TMEM101 | 2.613 |
|  |  |  | 49 | HLXB9 | 2.676 |
|  |  |  | 50 | FXYD3 | 2.311 |
|  |  |  | 51 | AR | 2.067 |
|  |  |  | 52 | SCGB2A1 | 3.967 |
|  |  |  | 53 | VTCN1 | 2.701 |
|  |  |  | 54 | CA8 | 2.107 |
|  |  |  | 55 | CB959193 | 3.930 |
|  |  |  | 56 | SPATA18 | 2.066 |
|  |  |  | 57 | C4BPA | 3.627 |
|  |  |  | 58 | KIAA1505 | 2.107 |
|  |  |  | 59 | CCNA1 | 2.228 |
|  |  |  | 60 | THC2708803 | 2.150 |
|  |  |  | 61 | STXBP6 | 2.007 |
|  |  |  | 62 | UNQ473 | 2.884 |

^a^Cutoff levels: FDR < 0.001, q-value < 0.001, and fold change ≥ 2.000. Order of presentation is according to ranking in SAM analysis.

**Supplementary Table S5:** **Overview of primers employed for PCR for *CTCF* exon mutation scanning**

| **Target^a^** | **Direction** | **^b^Sequence (5’ – 3’)** | **PCR Size (bp)** |
| --- | --- | --- | --- |
| Exon 3 | F | ACGTA**TGTAAAACGACGGCCAGT**TGTATATTTTTATTTAGACATGCTTTGCTTTAA | 319 |
|  | R | CAGATCAGGAAACAGCTATGACCGCCACTGTGCCCTCCATTACTT |  |
| Exon 3 | F | ACGTA**TGTAAAACGACGGCCAGT**AGCAGTGTACAGATGGTGATGATGG | 384 |
|  | R | CAGATCAGGAAACAGCTATGACCAGTTCCCCTTGTTCTAGTGTCTCCA |  |
| Exon 3 | F | ACGTA**TGTAAAACGACGGCCAGT**ATGATATGCCACACCCTACCTTTG | 388 |
|  | R | CAGATCAGGAAACAGCTATGACCCACTGTGTTGTATGCTTATCCCAAAAT |  |
| Exon 4 | F | ACGTA**TGTAAAACGACGGCCAGT**AATCTTAACACTTTGAAACTCTGCAGC | 351 |
|  | R | CAGATCAGGAAACAGCTATGACCACAAAAAGGGCATCATAAGAAATAATAAA |  |
| Exon 5 | F | ACGTA**TGTAAAACGACGGCCAGT**AGGGTTCCAGTCTCATAGCAGTTCT | 329 |
|  | R | CAGATCAGGAAACAGCTATGACCCCTCATCTTAAGTCCGTTTGGGTAGTA |  |
| Exon 6 | F | ACGTA**TGTAAAACGACGGCCAGT**GCTAAGCTTTTGTGCCTAACCTACTG | 364 |
|  | R | CAGATCAGGAAACAGCTATGACCGAATTCAGAGGATATGCCGGAGA |  |
| Exon 7 | F | ACGTA**TGTAAAACGACGGCCAGT**CATATCTGCCACCTGAGTTACCCTC | 364 |
|  | R | CAGATCAGGAAACAGCTATGACCAAGTGACAGGCCATTCCTATACATAAAA |  |
| Exon 8 | F | ACGTA**TGTAAAACGACGGCCAGT**GAATCGAGAAATGTATTAGTAACTTGTTAAAATG | 292 |
|  | R | CAGATCAGGAAACAGCTATGACCTAACACCAGACACCGAGAAAGCAC |  |
| Exon 9 | F | ACGTA**TGTAAAACGACGGCCAGT**CCTTTCCCCCTATGCCGTTTC | 341 |
|  | R | CAGATCAGGAAACAGCTATGACCGATGACTAACCCTTTCCAAACAATG |  |
| Exon 10 | F | ACGTA**TGTAAAACGACGGCCAGT**CCATTTCCCCTGAGCAGAGACA | 384 |
|  | R | CAGATCAGGAAACAGCTATGACCCCCGCCTCTATATCCCAGCAT |  |
| Exon 11 | F | ACGTA**TGTAAAACGACGGCCAGT**TCTTTCATCTTCCACCACCCTTCT | 322 |
|  | R | CAGATCAGGAAACAGCTATGACCGGCTTAGTTCCCACATCCCCA |  |
| Exon 12 | F | ACGTA**TGTAAAACGACGGCCAGT**TCCCGTTCGCTGTCAGTCTAAAA | 359 |
|  | R | CAGATCAGGAAACAGCTATGACCGCCAAAAAAAGAAAGAAGGGAGAAA |  |

^a^Exon 3 was sequenced in three overlapping regions due to size. ^b^Oligonucleotides were designed to contain M13F sequence 5`-TGTAAAACGACGGCCAGT-3` (bold) or M13R 5`-CAGGAAACAGCTATGACC-3` sequence (underlined) for subsequent direct sequencing, respectively.
